# A Shift from a Pivotal to Supporting Role for the Growth-Associated Protein (GAP-43) in the Coordination of Axonal Structural and Functional Plasticity

**DOI:** 10.3389/fncel.2017.00266

**Published:** 2017-08-31

**Authors:** Matthew R. Holahan

**Affiliations:** Department of Neuroscience, Carleton University Ottawa, ON, Canada

**Keywords:** GAP-43, protein kinase C, axons, development, regeneration, long-term potentiation

## Abstract

In a number of animal species, the growth-associated protein (GAP), GAP-43 (aka: F1, neuromodulin, B-50, G50, pp46), has been implicated in the regulation of presynaptic vesicular function and axonal growth and plasticity via its own biochemical properties and interactions with a number of other presynaptic proteins. Changes in the expression of GAP-43 mRNA or distribution of the protein coincide with axonal outgrowth as a consequence of neuronal damage and presynaptic rearrangement that would occur following instances of elevated patterned neural activity including memory formation and development. While functional enhancement in GAP-43 mRNA and/or protein activity has historically been hypothesized as a central mediator of axonal neuroplastic and regenerative responses in the central nervous system, it does not appear to be the crucial substrate sufficient for driving these responses. This review explores the historical discovery of GAP-43 (and associated monikers), its transcriptional, post-transcriptional and post-translational regulation and current understanding of protein interactions and regulation with respect to its role in axonal function. While GAP-43 itself appears to have moved from a pivotal to a supporting factor, there is no doubt that investigations into its functions have provided a clearer understanding of the biochemical underpinnings of axonal plasticity.

## GAP-43 Historical Overview

The growth-associated protein (GAP), GAP-43, has been known under various designations (see below) over the span of 40+ years in the course of separate yet overlapping investigations into phosphoproteins that contribute to plasticity and growth of the presynaptic terminal. Exploring this history has revealed the basic properties of this growth- and plasticity-related protein that set the foundation for work to determine whether this protein was a choke point for axonal malleability during states of plasticity or a component of a larger presynaptic proteome. From this historical perspective, it is clear that this presynaptic phosphoprotein of 43 kDa generated some excitement as it rose to the forefront of other polypeptides that were also under investigation. However, it also became clear that this protein functioned as a cog in a larger set of proteins and kinases to coordinate changes in axonal structure and function during times of plasticity.

### Growth-Associated Polypeptides

In 1974, a 43 kDa polypeptide was one among seven polypeptides noted to be present in the rabbit optic tract 8 days (but not earlier) following intraocular injection of [^35^S] methionine suggesting a slow transport rate of between 2 mm/day and 4 mm/day for these proteins (Willard et al., 1974). This 43 kDa polypeptide showed up again in the distal optic tract of both the guinea pig and rabbit 8 days following intraocular injection and was considered to be in the same group (Group IV based on axonal transport velocities) as actin and two forms of myosin-like polypeptides (Levine and Willard, 1980). The group IV polypeptides (bands 39, 43, S8, S29T, S13, S15 and S17 as noted in the article) were also prominent in the optic nerve of the toad preparation 8 days after injection (Skene and Willard, 1981b,d). This group contained at least 13 protein bands that were similar in the rabbit and toad and comprised the major fraction of labeled transported protein associated with actin (Willard et al., 1979; Skene and Willard, 1981b,d). Polypeptide 43 was found to be labeled in a temporal sequence similar to that of actin but disappeared more rapidly than actin in the optic nerve and optic tract (Willard et al., 1979). The authors suggested that the presence of polypeptide 43 in their sample may have been due to an interaction with bound actin (Willard et al., 1979). While there were a number of polypeptides associated with the actin-associated group IV polypeptides, these reports appear to be the first that initiated a series of experimental reports exploring the function of the 43 kDa polypeptide. However, this series of studies hinted that a diverse group of proteins was involved in axonal plasticity and it might be the sum of the functional properties of all the proteins, rather than one, that coordinated axonal dynamics.

Skene and Willard (1981b) detected rapidly transported polypeptides, referred to as GAPs of molecular weights of 24, 43 and 50 kDa, that showed a 20-fold increase in labeling over baseline following optic nerve crush in the frog. They showed that GAP-43, in particular, was transported in retinal ganglion cell axons of neonatal animals with a rapid decline during later stages of development highlighting a pattern of high expression during development and reduced expression during adulthood (Skene and Willard, 1981a). It was also shown that the GAP-43 protein was elevated after axotomy of the adult hypoglossal nerve, which regenerates, but was not induced by nonregenerating optic nerve injury in the adult (Skene and Willard, 1981a). In a third article by Skene and Willard (1981c), they reported on additional characteristics of three polypeptides that showed significantly elevated labeling during the regeneration of toad optic nerves. The three growth-associated polypeptides (GAP-24, GAP-43 and GAP50) showed strong associations with the plasma membrane and were judged to contain segments that extended away from the membrane (Skene and Willard, 1981c). GAP-50 (half-life = ~1 day) and GAP-43 (half-life = ~2–3 days) appeared to be transported preferentially to axon tips while GAP-24 (half-life = ~4–6 h) was distributed more uniformly along the axons. The authors offered the “GAP hypothesis” whereby these proteins would mediate functions critical for axon growth such that increased presence of any of these GAPs in a few neurons would be reflective of those neurons undergoing some form of axon growth (Skene and Willard, 1981a,b). They further suggested that regeneration would mimic an earlier development period of the neuron and that a number of GAPs (including GAP-43) would be involved in axon elongation (Skene and Willard, 1981a). Because GAP-43 showed a high concentration in the terminal portion of the axon, it was hypothesized to serve a critical link between the internal environment of the growing axonal tip and the intercellular environment (Skene and Willard, 1981c). However, once again, while GAP-43 was highlighted, the evidence suggested that it may be one of several proteins that subserves axonal plasticity.

### The Synaptic Phosphoprotein B-50

While studying the effects of adrenocorticotropic hormone (ACTH) on the phosphorylation of synaptic plasma membrane proteins *in vitro*, Zwiers et al. (1976) found that phosphorylation of a protein located at band 5 (48 kDa; among additional proteins located at bands 1 and 6–10) was insensitive to cAMP while bands 6–10 showed decreased phosphorylation in the presence of ACTH. In a methodological article examining the phosphorylation of separated synaptic plasma membrane proteins, the use of densitometric scanning with higher resolution resulted in the identification of more phosphoprotein peaks and a renumbering of protein bands (Wiegant et al., 1978). In this report, the scans were divided into four areas (A–D) with the main peaks numbered in tens. Therefore, the peak previously designated “5” (Zwiers et al., 1976) was designated B-50 (Wiegant et al., 1978). The phosphorylation of B-50 was shown to be inhibited by ACTH via an interaction with protein kinase activity in proximity to the membrane (Zwiers et al., 1978, 1979). A number of studies followed (see, Zwiers et al., 1980a,b) that examined the upstream kinase/phosphorylation aspects of B-50 as well its downstream effects on the phosphorylation of lipid membranes (Jolles et al., 1980). Protein kinase C (PKC) was shown to phosphorylate B-50 (Aloyo et al., 1982a,b) in a calcium-dependent fashion (Aloyo et al., 1983). Several articles examining the localization of synaptic membrane phosphoproteins found B-50 to be localized exclusively to nervous tissue (as opposed to a variety of peripheral organs; (Kristjansson et al., 1982) and within nervous tissue, to low density presynaptic membranes (Oestreicher et al., 1981; Sorensen et al., 1981; Gispen et al., 1985). These fundamental properties of B-50 were very similar to those described for GAP-43 (as above) but also showed that B-50 was not alone in its existence in the presynaptic compartment.

### CyclicAMP-Independent Phosphoprotein F1

In 1974, Ehrlich and Routtenberg (1974) reported on the properties of three phosphoproteins from the cerebral cortex that they termed D, E and F. B and F showed the highest level of endogenous phosphorylation but the phosphorylation status was not changed by addition of cAMP (Ehrlich and Routtenberg, 1974). Because Ehrlich and Routtenberg (1974) used the crude synaptosome preparation, their samples would have included the necessary enzymes and proteins (such as protein kinases) required for the actions of cAMP to facilitate the phosphorylation of a wide range of substrates (Bai and Witzmann, 2007; Kamat et al., 2014). In a follow-up study, protein component F (estimated to be 47 kDa) was shown to be the least sensitive to the effects of cAMP but attained the highest level of phosphorylation in the absence of cAMP (Routtenberg and Ehrlich, 1975) indicative of “some unique function for protein F” (p. 426; Routtenberg and Ehrlich, 1975). Modification of the phosphorylation status of band F and a composite band H-1 (estimated molecular weights 47 kDa and 10–18 kDa, respectively) were shown to be significantly increased 24 h after footshock and/or learning to escape from a footshock but minimally stimulated by the addition of cAMP (Ehrlich et al., 1977). The authors suggested the involvement of these proteins in some aspect of memory because the phosphorylation of F and H-1 was greater in the animals that had escaped footshock than in animals that experienced inescapable footshock (Ehrlich et al., 1977). In a similar, follow-up study, rats that were shocked and rats that were trained to escape footshock showed an elevation in phosphorylation activity of protein bands F1 (41 kDa) and F2 (47 kDa) from the frontal cortex (Routtenberg and Benson, 1980). While band F2 was the only protein to show significantly elevated shock-associated elevation in phosphorylation status, F1 showed the greatest variability leading the authors to suggest that the variability may be related to not only procedural aspects but also the *in vivo* state of the enzymes at the time of tissue assessment (Routtenberg and Benson, 1980). While these experiments showed a tight relationship between protein F1 (aka, GAP-43) its phosphorylation status and potential memory function, there was no direct evidence for its role in axonal plasticity bridging these functions.

In a report examining other factors that might influence the *in vivo* phosphorylation state of protein F1, handling was shown to significantly reduce F1 phosphorylation in the hippocampus compared to non-handled rats (Cain and Routtenberg, 1983). While this finding by itself is of interest, the introduction section of the article makes reference to the likelihood that protein F1 is the same as B-50. The Routtenberg lab continued to characterize their protein F1 with respect to axonal plasticity (references below) and Gispen et al. (1986) published a report to sanctify the similarity between protein F1 and B-50. In the discussion of this article, the authors described how protein F1/B-50 shared many characteristics with GAP-43, GAP-48 and pp46.

### Growth-Associated Protein (GAP)-43, GAP-48, Protein 4, B-50, F-I, y5 and pp46

In a fourth line of research, an optic nerve crush model in fish was used to examine changes in protein synthesis in retinal ganglion cells during various stages of axonal regeneration (Benowitz et al., 1981). Eight days after optic nerve crush, the strongest signals were noted for proteins with molecular weights of 24–27, 44 and 210 kDa (Benowitz et al., 1981). The proteins in the range of 44–49 kDa were shown to not only increase during the early stages of regeneration but also increase independently from postsynaptic signals arising from the tectum (Benowitz and Lewis, 1983; Yoon et al., 1986). These proteins (44–49 kDa) were present in both the membrane-bound and soluble fractions of material transported from the retinal ganglion cells to the nerve terminals in the tectum. During regeneration, their labeling increased up to 100-fold reflecting new protein synthesis and an increase in total amount of protein present in the sample (Benowitz and Lewis, 1983; Benowitz et al., 1983). Following up on this work, an acidic, 48 kDa, membrane-bound protein (GAP-48) showed a 50- to 100-fold increase in retinal ganglion cells undergoing regeneration (Perrone-Bizzozero and Benowitz, 1987). Once again, rather than only one protein being the sole factor in axonal regeneration, it seemed rather that a number of proteins worked together to coordinate appropriate regenerative responses.

The discussion of this article appears to be one of the first to compare GAP-48 and B-50 concluding that these proteins are likely homologous (Perrone-Bizzozero and Benowitz, 1987). The authors also described that B-50 was identical to GAP-43 (verified by Jacobson et al., 1986) and the same protein designated as pp46 (Perrone-Bizzozero and Benowitz, 1987). A link was also described for B-50 and F1 suggesting that these proteins (GAP-48, GAP-43, B-50, F1, pp46) performed the same function in playing a role in the initial development of neural relationships and subsequent modulation as would occur during regeneration and long-term potentiation (LTP; Perrone-Bizzozero and Benowitz, 1987; Moya et al., 1988). The variations in molecular weights of these various proteins was likely in large part due to technical aspects of the assays (Benowitz et al., 1987) and the likelihood that GAP-43/ B-50 protein existed as a complex with PKC and phosphatidylinositol phosphate (PI3)-kinase (Zwiers et al., 1980b; Jacobson et al., 1986; Nguyen et al., 2009).

### Neuromodulin

In 1983, Andreasen et al. (1983) characterized a protein they termed P-57 that showed a strong affinity for calmodulin (CaM) during conditions of low Ca^2+^ binding but CaM dissociated from P-57 during conditions of high Ca^2+^ presence. They proposed that P-57 may function to increase CaM concentrations near the membrane and release it with elevations in intracellular Ca^2+^ concentrations (Andreasen et al., 1983). P-57 was found to be the most abundant CaM-binding protein specifically located in the brain, spinal cord and retina but no other tissues (Cimler et al., 1985). Within these neural tissues, it was found in both membrane (white matter) and soluble (cell body) fractions (Cimler et al., 1985). P-57 was estimated to have a molecular weight of 25.7 kDa and the P-57-CaM complex was estimated to have a molecular weight of 45.6 kDa (Masure et al., 1986). The Ca^2+^-dependent PKC phosphorylated P-57 at a serine residue but P-57 was not phosphorylated by the cAMP-dependent protein kinase (Alexander et al., 1987). An interesting relationship between the binding of CaM and PKC to P-57 was then determined such that CaM decreased the rate of P-57 phosphorylation by PKC and PKC phosphorylation prevented P-57 binding to CaM suggesting CaM may be liberated from P-57 via rises in intracellular Ca^2+^-dependent PKC phosphorylation (Alexander et al., 1987). The P-57 sequence contained a hydrophilic amino acid composition but lacked a hydrophobic segment that would indicate an interaction with the membrane (Wakim et al., 1987). The neurospecific CaM-binding protein was then renamed neuromodulin (Alexander et al., 1988) and was determined to be similar to p-57, GAP-43, B-50 and F1 (Baudier et al., 1989).

### Summary

Seemingly, five independent lines of research came to complementary and overlapping conclusions on the fundamental properties of an axonal-associated membrane-bound protein related to axonal plasticity. The varied molecular weights reported for the different protein terminologies were noted by Benowitz et al. (1987) who discussed technical aspects such as gel composition or the presence or absence of detergent that might contribute to the differential weights. Other contributing factors to the weight differences were suggested to be the status of the protein when run on the gels such as phosphorylated or bound by CaM, or whether it was bound with other membrane proteins. Nevertheless, converging lines of evidence indicated that these varied observations were of a protein with the same function. There were also independent observations from each line of work that stimulated in-depth investigations in to the molecular and biochemical aspects of this protein and its potential contribution to axonal outgrowth. However, it must be kept in mind that through these five foundational lines of research, there were a number of other proteins that showed similar properties with respect to axonal outgrowth and it might be concluded that no one protein was a pivotal factor but that all proteins played a supporting role in axonal plasticity—including GAP-43. While the author of this review concludes that the transcriptional, post-transcription and post-translational regulation of numerous proteins coordinates axonal plasticity, the work that explored the contribution of GAP-43 to axonal outgrowth has been instrumental in elucidating basic mechanisms of axonal growth and function.

## GAP-43 and Presynaptic Plasticity

Since those reports, interest in GAP-43 (the nomenclature for this protein used in the current review) rose sharply peaking at 134 PubMed reports in 1995 but showing a decreasing trend over the past 20 years (lowest in 2016 at 65 publications). It appears that this protein was initially hailed as playing a pivotal role in axonal outgrowth but has since become a contributing factor as part of a larger group of axonal growth-permissive proteins. The study of GAP-43 has revealed a tight relationship between its expression and axonal structural plasticity during development, a rise in its expression levels following axonal injury during the regeneration phase and an association with input-dependent neuroplastic processes, such as LTP and memory formation, in conjunction with reorganization of axonal terminal fields (Denny, 2006). The functional attributes of GAP-43 can be ascribed to the transcriptional and post-transcriptional regulation of the mRNA and the protein-kinase C-dependent post-translational modification (phosphorylation) and protein localization at the biochemical level. Many growth permissive events in the central nervous system lead to elevations in GAP-43 so, while it may have moved from a pivotal role to a supporting role, it is no doubt an important node in the protein network involved in axonal function. A review of the transcriptional and translational aspects of GAP-43 as they pertain to the moderation of neuroplastic events in the central nervous system will be examined in relation to the function of this protein as an important node in axonal outgrowth during development and regeneration following injury.

## GAP-43 Gene Characteristics

### Gene and Transcriptional Regulation

**Figure 1:**
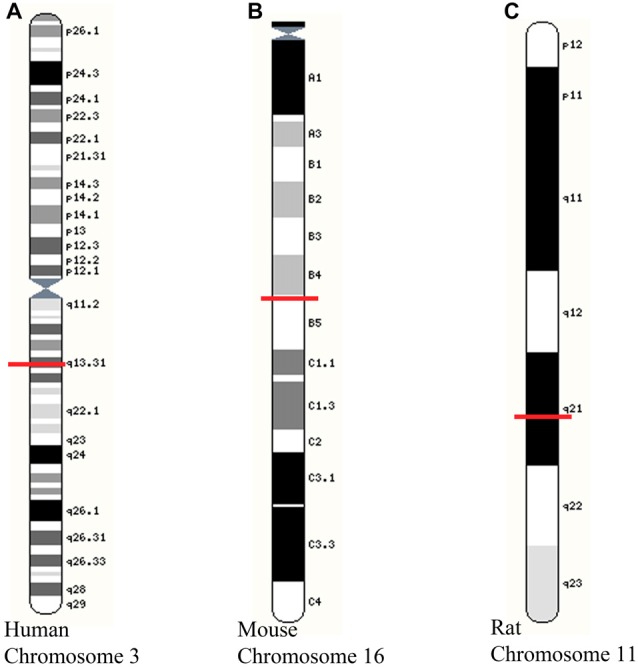
Location of growth-associated protein (GAP)-43 gene on chromosomes of different species. **(A)** Human chromosome 3 showing GAP-43 gene at locus 3q13.31; **(B)** Mouse chromosome 16 showing GAP-43 at 16 B4; 16 28.37 cM; **(C)** Rat chromosome 11 showing GAP-43 gene at 11q21. Images adapted from Yates et al. (2016). Red line indicates position of GAP-43 gene for each species and chromosome.

Expression of GAP-43 mRNA is restricted to the nervous system, and in particular, axons, where levels increase during periods of neurite outgrowth (Karns et al., 1987). Developmental and regeneration-associated changes in GAP-43 synthesis appear to be mediated via transcription of the same, single gene (Basi et al., 1987). The human and rat GAP-43 genes are thought to be expressed in similar ways due to the existence of two similar transcripts, a high amount of overlap between the sequences, and promoter activities (Nedivi et al., 1992; Ortoft et al., 1993; de Groen et al., 1995; Udvadia et al., 2001). The human GAP-43 gene has been localized to chromosome 3 while the mouse GAP-43 gene is localized to chromosome 16 (Kosik et al., 1988) and the rat GAP-43 gene localized to chromosome 11 (Figure 1). On the rat GAP-43 gene, there are proximal (E1 and E2) and distal (E3 to E7) clusters of E-boxes (Chiaramello et al., 1996). The regulation of GAP-43 expression occurs via a basic helix-loop-helix mechanism acting on the E1 E-box positioned in active P2 promoter that is neuronally restricted. The E1 E-box is the only E-box that is conserved between the rat and human GAP-43 promoter regions with respect to core sequence, flanking sequence, and position (Chiaramello et al., 1996). The GAP-43 gene contains two promoters: P1 consists of promoter elements such as TATA and CCAAT boxes (Nedivi et al., 1992; Ortoft et al., 1993) while P2 does not encompass either of these elements (Eggen et al., 1994; Weber and Skene, 1997). Within the GAP-43 gene is a repressive element (also found on SNAP-25 and nNOS genes aptly named “SNOG”) found downstream of the TATA region that contributes to the neuron specificity of gene expression by inhibiting transcription in a variety of non-neuronal cells and tissues (Weber and Skene, 1997, 1998). The majority of GAP-43 mRNA is derived from the P2 promoter in both P19-EC cells and 8-day old rat brain (Eggen et al., 1994) suggesting that the P2 promoter may play a more prominent role in regulation of outgrowth.

The GAP-43 gene includes three exons in both the rat (Grabczyk et al., 1990) and human (Nielander et al., 1993). The first exon encodes the N-terminal 10 amino acids of the membrane-targeting domain; the second exon encodes the CaM-binding domain and PKC phosphorylation site; exon 2 in humans includes a 10 amino acid residue insert not found in rodents (Grabczyk et al., 1990). These gene characteristics confirm the earlier studies showing the CaM and PKC post-translational modification domains.

### Post-Transcriptional Regulation

Modulation of the degradation rate of GAP-43 mRNA appears to be a main factor in the control of mRNA levels during neurite outgrowth (Perrone-Bizzozero et al., 1991). Nerve growth factor (NGF) and phorbol ester (TPA) have been shown to selectively stabilize GAP-43 mRNA in PC12 cells (Perrone-Bizzozero et al., 1993). PKC activity appears to regulate GAP-43 mRNA in a translation-independent mechanism as inhibition of PKC, but not treatment with cycloheximide, can prevent NGF- and TPA-induced stabilization of GAP-43 (Perrone-Bizzozero et al., 1993). The ELAV-like protein 4 (HuD; Szabo et al., 1991; Sanna et al., 2014b), a neuronal RNA-binding protein (Mobarak et al., 2000), appears to be a main stabilizing agent for GAP-43 mRNA. Events associated with elevated HuD expression include times of neural development, during the course of nerve regeneration, and episodes of stimulus-dependent modification such as memory functions (Deschênes-Furry et al., 2007). Based on this, HuD has been hypothesized to be important for prolonging gene stability and, as such, would be instrumental in sustaining elevated levels of mRNA species associated with axonal outgrowth including GAP-43 mRNA (Perrone-Bizzozero and Bolognani, 2002). Experimental evidence has shown prolonged stability of GAP-43 mRNA in neural tissue from mice expressing elevated levels of HuD via transgenic means compared to non-transgenic littermates supporting the contention that HuD can positively affect GAP-43 mRNA stability (Bolognani et al., 2006). As well, transgenic overexpression of HuD was associated with an increase in GAP-43 mRNA expression in granule cells of the hippocampal dentate gyrus, neurons in the lateral amygdala and layer V neurons of the cortex (Bolognani et al., 2006). Inhibition of PKC prevents HuD and GAP-43 overexpression and decreasing levels of PKCγ and HuD were associated with GAP-43 reductions (Sanna et al., 2014a). The HUD-dependent stabilization of GAP-43 mRNA or other mRNAs involved in axonal outgrowth could prove to be key elements in enhancing axonal growth and regeneration. In essence the continued work in identifying key transcriptional and post-transcriptional regulators of a larger set of regeneration-associated genes, rather than individual genes like GAP-43, lends support to the conclusion that a number of axonal proteins function together to mediate plasticity.

## GAP-43 Protein

### Translational and Post-Translational Characteristics

**Figure 2:**
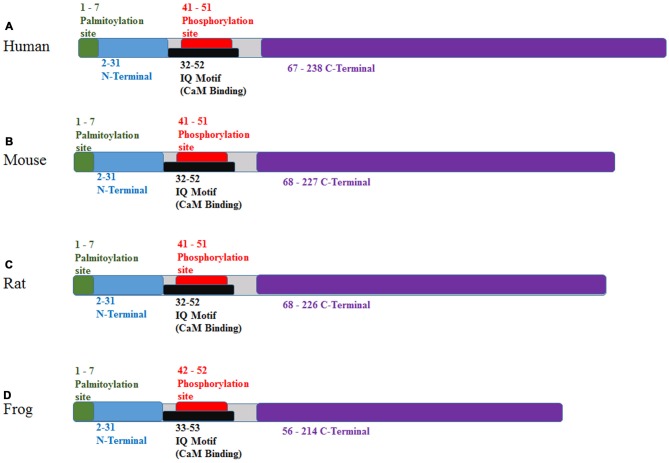
Protein binding domains of GAP-43 for various species. **(A)** Human, **(B)** mouse, **(C)** rat and **(D)** frog. Sequences as found at http://www.ebi.ac.uk/interpro/protein/P06837/similar-proteins. Additional details comparing individual amino acid sequences for human, mouse and rat can be found at http://www.phosphosite.org/proteinAction?id=2142&showAllSites=true.

In humans, GAP-43 is 238 amino acids, while in the mouse, it is 227; in the rat, 226 and in the frog, 214 amino acids (see Figure 2). The hydrophilic protein (Chan et al., 1986) is encoded by a 1.5 kb brain-specific, developmentally-regulated mRNA (Rosenthal et al., 1987) and lacks a membrane-spanning domain and contains no sites for glycosylation (Basi et al., 1987). Analysis has revealed a short hydrophobic amino acid sequence segment which functions to anchor the GAP-43 protein on the cytoplasmic side of the presynaptic plasma membrane (Basi et al., 1987; Gorgels et al., 1989). Localization of GAP-43 protein to the inner surface of the plasma membrane of growing axons, including growth cones, is based on palmitoylation of Cys3 and Cys4 (Strittmatter et al., 1994; Kong et al., 2013; Tuodziecka et al., 2013). Primary palmitoylation takes place at the endoplasmic reticulum-Golgi intermediate compartment (McLaughlin and Denny, 1999). A reduction in the palmitoylation of GAP-43 and other growth-related proteins is sufficient to stop advancing axons during critical remodeling periods and only at the close of these remodeling periods, does the availability of these proteins (including GAP-43) decline (Patterson and Skene, 1999) suggesting other post-translational modifications take over to stabilize or fine tune synaptic terminals. In this case, palmitoylation serves to post-translationally modify GAP-43 for transport to the membrane then local phosphorylation follows to regulate protein mobility and plasma membrane targeting of GAP-43 (Gauthier-Kemper et al., 2014).

Phosphorylation of GAP-43 importantly contributes to the biochemical and physiological activities of the protein with Ser41 being the singular target of PKC-regulated phosphorylation, (Meiri et al., 1988; Coggins and Zwiers, 1989; Yi et al., 2011) specifically, PKCbeta (Rosenthal et al., 1987; Sheu et al., 1990b). With low Ca^2+^ levels in the presynaptic terminal, CaM shows a high affinity for GAP-43 and when Ca^2+^ levels rise, as during high plasticity events, CaM is released (Neve et al., 1998). If the Ser41 site is transformed from serine to aspartate with the outcome of mimicking constitutive phosphorylation, binding of CaM to GAP-43 is absent (Chao et al., 1996). CaM binding can entirely prevent PKC-induced phosphorylation of GAP-43 so serves to quell the activity of GAP-43 during periods of low activity in neurons (Chan et al., 1986; Kumar et al., 2013). If Ser41 is mutated from serine to alanine (S41A), phosphorylation is prevented and there is a discernible reduction in presynaptic branching and growth and, indeed, only half of brain/spinal neurons expressing S41A successfully initiate axonal outgrowth (vs. nearly 100% for wild type GAP-43; Leu et al., 2010). This shows the importance of the amino acid sequence that flanks the Ser41 phosphorylation site in the promotion of outgrowth and may be due, in part, to a liberation of CaM and interactions with PIP2 (see below). The results of this report also indicated that the PKC-GAP-43 pathway contributes to an F-actin based process in the stabilization of new synapses and initiation of new terminal branches (Leu et al., 2010). Therefore, changes in the phosphorylation status of GAP-43 is a critical factor in its contribution to axonal outgrowth. Equally important is the liberation of CaM upon phosphorylation, which may be a pivotal factor in mediating axonal outgrowth via interactions with the presynaptic proteome. In this respect, GAP-43 would serve to liberate the pivotal factor (CaM) thereby serving a permissive role in axonal outgrowth mechanisms.

In the absence of Ca^2+^, arachidonic acid (AA) imparts a marginal effect on GAP-43 phosphorylation (Schaechter and Benowitz, 1993). AA, a retrograde stabilizing factor, is released by cytoplasmic phospholipase A2 (cPLA2) after Ca^2+^ entry through activated post-synaptic N-methyl-D-aspartate receptors (NMDARs) and can activate presynaptic PKC to phosphorylate various substrates such as GAP-43 to regulate cytoskeletal dynamics (Leu and Schmidt, 2008; Leu et al., 2010) directly or via the release of CaM. Because of these NMDAr-mediated retrograde responses, NMDAr’s have been functionally associated with the GAP-43 protein even though NMDAr’s are postsynaptically located and GAP-43 has a presynaptic location. When Ca^2+^ levels rise to those that mimic levels in the nerve terminal during synaptic activity, AA works synergistically with Ca^2+^ to increase the sensitivity of GAP-43 phosphorylation and bump up the maximal phosphorylation level by 50% (Schaechter and Benowitz, 1993). The augmenting endpoint of the synergistic actions of AA and Ca^2+^ is mediated by PKC-induced phosphorylation (Schaechter and Benowitz, 1993). In the optic nerve preparation, AA induces NCAM promotion of axon outgrowth by activating PKC-induced phosphorylation of presynaptic GAP-43 and ultimately, the stabilization of F-actin (Schmidt et al., 2004).

### Protein Interactions

An important consideration in determining that GAP-43 is a supporting factor in axonal outgrowth and function is through the examination of protein interactions (see Holahan, 2015 for summary diagrams). When GAP-43 is phosphorylated by PKC following rises in intracellular Ca^2+^, it interacts with other proteins to facilitate axonal elongation and vesicular cycling. One functional outcome of PKC-induced phosphorylation of GAP-43 during activity-dependent increases in presynaptic Ca^2+^ levels is the modulation of synaptic vesicle recycling (Cousin, 2000). The facilitation of exo- and endocytotic processes likely occurs through interactions with synaptophysin (Verkade et al., 1996; Hou and Dahlström, 2000), SNAP-25 (Goutan et al., 1999; Rekart and Routtenberg, 2010) and rabaptin-5 (involved in membrane fusion and membrane trafficking of recycled endosomes; Neve et al., 1998; Chia et al., 2013). In its service to vesicle recycling, GAP-43 augments extracellular signals through interactions with the GTP-binding protein, Go, enhancing the sensitivity of Go and altering the predilection for neuronal outgrowth (Strittmatter et al., 1991, 1993, 1994). A second outcome of PKC-induced phosphorylation of GAP-43 is the liberation of CaM, which can then carry out its own independent functions (not reviewed in detail here but for reviews, see McCue et al., 2010; Naz et al., 2016). With the liberation of CaM from phosphorylated GAP-43, it interacts with CaMKII resulting in the phosphorylation of a number of substrates including cofilin by LIM kinases, which result in neurite outgrowth (Arber et al., 1998; Heng and Koh, 2010).

## GAP-43 Localization

### Cellular Compartmentalization

The neuron-specificity of GAP-43 is highlighted with its high density in presynaptic terminals in both the peripheral and central nervous systems. In neuroglial cells, GAP-43 shows a plasma membrane localization in neonatal rat cortical astrocytes (Vitković et al., 1988; Vitković and Mersel, 1989; da Cunha et al., 1991) as well as oligodendrocytes and type 2 astrocytes (Deloulme et al., 1990). In neuronal cells, GAP-43 is distributed throughout all neural compartments but shows the highest density in axon terminals including growth cones (Donnelly et al., 2013; Yoo et al., 2013). GAP-43 is largely absent from dendrites and myelinated axons as shown by a lack of double labeling with microtubule-associated protein 2 (MAP-2) and the large neurofilament proteins (Ramakers et al., 1992). Double-immunohistochemical labeling in cultured hippocampal neurons shows that GAP-43 colocalizes with the axonal marker Tau with the strongest signal at axonal puncta of developing neurons (Morita and Miyata, 2013). During the period of neurite extension in cultured neurons, the soma shows reduced GAP-43 staining and the growing tips of the axons (i.e., growth cones) show increased, punctate GAP-43 staining (Meiri et al., 1988). The growth-cone localized GAP-43 is associated with the inner membrane surface as shown by positive immunostaining after permeabilization of the membrane (Meiri et al., 1988). With respect to its localization to neurons, there was an absence of GAP-43 labeling in Schwann cells and fibroblasts (Meiri et al., 1988). These data point to a strong association between the presence of GAP-43 and outgrowth of axons.

Due to its cellular localization, GAP-43 more than likely contributes to axon terminal structural reorganization. In this respect, GAP-43 has been shown to promote F-actin accumulation as occurs following stimulus-induced nerve sprouting at the neuromuscular junction (Frey et al., 2000). During axonal structural modification, GAP-43 accumulates at subplasmalemmal rafts on the inner membrane where it adjusts F-actin accumulation via interactions with PI(4,5)P_2_ (Laux et al., 2000). Down-regulation of GAP-43 function with an *in vivo* lentiviral-mediated gene silencing technique resulted in the degeneration of climbing fibers in the olivo-cerebellar system as assessed with a decrease in length, branching and number of synaptic boutons (Grasselli et al., 2011) showing a causal relationship between GAP-43 levels in mediating this outgrowth.

### Neuroanatomical Localization

GAP-43 is widely expressed in the central nervous system during the perinatal period with subsequent reduced levels as maturation progresses. In the mature central nervous system, GAP-43 levels remain high in structures likely to undergo input-dependent plasticity including the cerebellum (granule cells (Casoli et al., 2001) but not Purkinje cells (Meberg and Routtenberg, 1991)), neocortex, entorhinal cortex (Kruger et al., 1992), hippocampus, olfactory bulb (McGuire et al., 1988; De la Monte et al., 1989) and retinal cells (Freeman et al., 2011). Significant levels of GAP-43 are also observed in adult-born olfactory sensory neurons (Schwob et al., 1992). The idea that GAP-43 may be of critical importance to growing axonal terminals is supported by reports showing the localization of GAP-43 expression in olfactory sensory neurons (Verhaagen et al., 1989; Holtmaat et al., 1999), which are generated throughout life. This finding allows a dissociation of axonal development from effects on axonal outgrowth and suggests an important contribution of GAP-43 to axonal growth no matter when or where it is expressed. Layer I neurons of the cortex and CA1 pyramidal cells of the hippocampus (Casoli et al., 2003) but not hippocampal granule cells (Meberg and Routtenberg, 1991; McNamara and Lenox, 1997) show dense GAP-43 labeling. Pronounced labeling of GAP-43 has also been found in a variety of adult subcortical structures such as the caudate-putamen, olfactory tubercle (Ramakers et al., 1992), nucleus accumbens, bed nucleus of the stria terminalis, amygdala and medial preoptic area of the hypothalamus (Benowitz et al., 1988). There is also some specificity of GAP-43 labeling overlap with specific neurotransmitter systems such as those in the substantia nigra pars compacta (dopamine), the locus coeruleus (norepinephrine), and dorsal raphe (serotonin; Bendotti et al., 1991; Meberg and Routtenberg, 1991; Kruger et al., 1993; Denny, 2006). The medial septum, nucleus basalis magnocellularis and the vertical limb of the diagonal band express intermediate GAP-43 levels, while the horizontal limb of the diagonal band and the substantia innominata express higher levels (McKinney and Kent, 1994).

Within the spinal cord and brainstem, unmyelinated or moderately myelinated areas, such as the substantia gelatinosa and the nucleus of the solitary tract, express high levels of GAP-43 (Kapfhammer and Schwab, 1994). GAP-43 staining is detected in small unmyelinated axons (0.12–0.15 microns diameter) and small (0.35 microns) axon terminals comprised of round vesicles that form asymmetric synapses with thin spines (DiFiglia et al., 1990). The localization of GAP-43 in these small myelinated and unmyelinated fibers is found in terminals that make single axodendritic or axosomatic synapses (Curtis et al., 1993). Within motor neurons of the brainstem and spinal cord, GAP-43 is present at all vertebral levels with higher concentrations in cervical and thoracic regions (Curtis et al., 1993; Berg et al., 2012; Gordon and Tetzlaff, 2015).

## Involvement in Developmental Outgrowth of Axons

GAP-43 is noted to be involved in developmental neurite outgrowth through the amplification of pathfinding signals from the growth cone (Strittmatter et al., 1995). In cultured neurons, GAP-43 labeling was clustered in growth cones 6 days after plating and after 10 days, cell body labeling was absent while at 20 days, the growth cone labeling was minimized (Burry et al., 1991). Transgenic expression of GAP-43 in mature olfactory sensory neurons, where GAP-43 is strongly expressed in immature olfactory sensory neurons, produced numerous olfactory axons with enlarged endings (Holtmaat et al., 1995). This effect demonstrates that reintroduction of a developmentally-regulated gene in neurons that no longer express this gene can recapitulate a development axonal growth process (Holtmaat et al., 1995). Developmentally-regulated expression of GAP-43 and associated axonal growth has been hypothesized to be controlled by activity-independent transcriptional processes and input-dependent, NMDA-receptor mediated posttranslational mechanisms (Cantallops and Routtenberg, 1999).

Numerous factors interact with GAP-43 during neurite outgrowth lending to the contention that GAP-43 may play a supporting role in the orchestration of axonal outgrowth. A marked increase in GAP-43 levels occurs in conjunction with the induction of a neuronal phenotype in PC12 pheochromocytoma cells by NGF (Irwin et al., 2002). Three hours after NGF exposure, GAP-43 levels rise and reach maximal levels 24 h after NGF application (Costello et al., 1990). In addition, NGF-induced responses in PC12 cells can be enhanced with upregulation of GAP-43 transfection, suggesting that NGF interacts with presynaptic GAP-43 to modulate neurite outgrowth (Neve et al., 1991). In cerebellar granule cells, NCAM-mediated fibroblast growth factor (FGF) activation leads to increased phosphorylation of GAP-43 and neurite outgrowth and, importantly, neither NCAM nor FGF stimulated neurite outgrowth when the GAP-43 gene was knocked-out (Meiri et al., 1998).

During the early phases of development, newly formed synapses show reduced levels of palmitoylated GAP-43 (Patterson and Skene, 1999) which may indicate a signal to stop advancing axons, suggesting a developmental switch for GAP-43 palmitoylation that is essential to disengage the molecular apparatus for axon extension (Patterson and Skene, 1999). Using a culture system, the morphological relationship between dorsal root ganglion (DRG) explants and dissociated skeletal muscle (SKM) cells was investigated to determine the influences on neurite growth and neuronal migration (Zhang and Li, 2013). The number of migrating neurons and the percentage of neurofilament (NF-200)- and GAP-43-positive neurons and their associated mRNAs increased significantly in neuromuscular cocultures (with SKM) compared to DRG explants alone suggesting target SKM cells promote neurite outgrowth and neuronal migration of DRG explants (Zhang and Li, 2013).

GAP-43 expression and functional outcomes during development appear to be brain region-dependent. In cerebellar granule cells, GAP-43 mRNA expression functions in differentiation and migration of neurons while in parallel and climbing fibers, GAP-43 contributes to axonal outgrowth and synaptogenesis (Console-Bram et al., 1996). In the cerebellum, GAP-43 mRNA expression increases from birth to postnatal day seven (PND 7) then declines during the ensuing sculpting and maturation processes (Console-Bram et al., 1996). By PND21, GAP-43 mRNA expression localizes to the internal granule layer and inferior olivary nuclei with little hybridization signal in deep cerebellar nuclei and absent expression in the molecular layer as seen in the adult (Console-Bram et al., 1996). In the optic nerve and optic fiber layer of the retina, GAP-43 staining was high at birth (PND0) and was absent between PND8 and PND16 (Kapfhammer et al., 1994). In auditory brainstem neurons, GAP-43 protein distribution is unmistakable in the cochlear nuclear complex subdivisions and the superior olivary complex at birth (PND0; Horváth et al., 1997). From PND8 to PND12, GAP-43 staining in these areas becomes punctate indicative of presynaptic ending formation. By PND16, the auditory brainstem nuclei are mostly devoid of GAP-43 staining with the exception of the presynaptic terminal puncta (Horváth et al., 1997). In cat primary visual cortex (VI), GAP-43 phosphorylation was elevated approximately 10-fold during the postnatal developmental period from PND1 to week 3 then showed a gradual decrease by an overall estimate of 2.5-fold by week 51 (Sheu et al., 1990a). In the cortex and hippocampus, quantification of GAP-43 protein was elevated during a period of elevated synaptogenesis from PND14 to PND21 (Morita and Miyata, 2013). Results from this study confirmed that GAP-43 is highly expressed in immature growing axonal terminals and decreases during maturation with labeling showing an inverse labeling pattern with synapsin and synaptotagmin (Morita and Miyata, 2013).

During the aging process, GAP-43 levels show age-related decrements. Both male and female aged rats exhibited less GAP-43 mRNA in the hippocampus than younger counterparts (Chao et al., 1992). *In vitro* phosphorylation of GAP-43 in the hippocampus of 5-, 11- and 25-month old rats revealed a near 50% reduction in the 25-month old rats when compared to 11-month old rats (Barnes et al., 1988). A decrease of GAP-43 immunoreactivity was also noted in the dentate gyrus, cingulate cortex and olfactory bulb in 31-month-old Wistar rats compared to 3- and 18-month olds (Casoli et al., 1996).

During development of the human brain, GAP-43 mRNA expression shows a sharp decline from birth to 2 years of age and levels-off until about 5 years of age (Webster et al., 2011). In contrast, levels of GAP-43 protein appear to remain similar across these ages (Webster et al., 2011). In Alzheimer’s disease (AD), PKC-mediated phosphorylation of GAP-43 has been shown to be decreased (Florez et al., 1991). End-stage AD brains exhibited reduced neuronal expression of GAP-43 mRNA with GAP-43 protein showing translocation to membranes of swollen neurites (de la Monte et al., 1995). Downregulated and ectopic GAP-43 gene expression were hypothesized to reveal a signature molecular indicator that preceded and progressed with synaptic degeneration and ensuing dementia in AD (de la Monte et al., 1995). In AD cases with high phosphorylated tau (tangle) density, GAP-43 mRNA expression was reduced fivefold compared to AD brains with low tangle density (Coleman et al., 1992). In addition, a decrease in GAP-43 mRNA was found in neurons showing tangle formation compared to adjacent neurons without tangles in the parahippocampal cortex of AD patients (Callahan et al., 1994). These correlations are worthwhile of further exploration to determine the nature of the relationship of these observations (i.e., whether tangle formation reduces GAP-43 expression, whether reduced GAP-43 causes tangle formation or whether both are a product of some other upstream change such as plaque formation).

## GAP-43 as A Moderator of Neuroplasticity

### Long-Term Potentiation

LTP represents a set of pre- and post-synaptic cellular responses that beget synaptic plasticity and may function to facilitate axonal outgrowth (Bourne et al., 2013) during development and regenerative processes after injury. GAP-43 is phosphorylated by a Ca^2+^-dependent PKC process that is critical for its functional contributions to LTP and synaptic plasticity (Akers and Routtenberg, 1985). *In vitro* GAP-43 phosphorylation was selectively increased 5 min after the induction of LTP and was specifically related to enhanced synaptic efficiency (as measured by an increase in the spike amplitude after high frequency stimulation compared to baseline) because low frequency, nonpotentiating stimulation did not increase (or decrease) GAP-43 phosphorylation (Routtenberg and Lovinger, 1985). Three days following perforant path LTP induction, dorsal hippocampus tissue showed augmented GAP-43 phosphorylation which was greater than tissue from control animals that received low frequency stimulation (Lovinger et al., 1985). *In vivo* tetanic stimulation of the mossy fiber pathway was associated with increased PKC-mediated GAP-43 phosphorylation one and five but not 60 min after stimulation indicative of a role for GAP-43 phosphorylation in the induction but not maintenance of LTP within mossy fiber synaptic membranes (Son et al., 1997). GAP-43 mRNA expression in hilar cells was increased after a 48-h time lapse following *in vivo* perforant path tetanus stimulation in the intact mouse (Namgung et al., 1997). The authors suggested that phosphorylation of GAP-43 in the axon terminals by PKC would set off a retrograde signal back to the nucleus to initiate transcription. Inhibition of PKC-induced phosphorylation of GAP-43 with monoclonal antibodies prevented the induction of LTP in CA1 pyramidal neurons *in vitro* (Fedorov et al., 1995). Ten to 60 min, but not 90 min, following LTP induction in the CA1 field of rat hippocampal slices, GAP-43 phosphorylation was increased (Ramakers et al., 1995) but elevated phosphorylation was not seen if stimulation failed to induce LTP (Ramakers et al., 1995, 1999). As well, one but not 2 h after LTP induction, GAP-43 and PKCγ mRNA hybridization were increased (Meberg et al., 1995) whereas 3 days after LTP induction, GAP-43, PKCβ and PKCγ mRNA expression in the CA3 subfield was decreased (Meberg et al., 1993). The authors hypothesized that decreased expression 3 days after LTP might be associated with a reduced growth potential leading to synaptic stabilization in the activated pathways (Meberg et al., 1993).

Genetic overexpression of the constitutively phosphorylated form of GAP-43 enhanced presynaptic paired-pulse facilitation and LTP in the CA1 hippocampal region (Hulo et al., 2002); an effect that was absent in transgenic mice overexpressing the non-phosphorylatable form. The authors concluded that the phosphorylation state of GAP-43 facilitated presynaptic function affecting LTP and, ultimately, synaptic plasticity (Hulo et al., 2002). As mentioned, presynaptic GAP-43 phosphorylation can be augmented in a PKC-dependent manner by retrograde messengers liberated from postsynaptic NMDA receptor activation (Luo and Vallano, 1995). Application of AA at LTP-inducing concentrations significantly increased translocation of PKC to the presynaptic membrane in hippocampus with concomitant increased GAP-43 phosphorylation suggesting that AA contributes to LTP via activation of presynaptic PKC-mediated phosphorylation of GAP-43 (Luo and Vallano, 1995).

### Memory Formation

Because LTP induction is associated with increased phosphorylation of GAP-43, it is important to demonstrate that similar changes in GAP-43 phosphorylation occur with measures of behavioral memory. In this vein, heterozygous knock-out of GAP-43 in mice resulted in a compromised memory for a shock-paired environment (Rekart et al., 2005). This was specific to a contextual memory as there were no deficits in shock-conditioning to a tone cue and tests of nociceptive and auditory function were intact (Rekart et al., 2005). In a related study, phosphorylation of hippocampal GAP-43 remained elevated for 1.5–72 h after acquisition of a context-shock association (Young et al., 2002). Also noted in this study was that 15–90 min after context-shock acquisition, PKCα and PKCγ translocated to the membrane, while 15–30 min after training, PKCβII and PKCε translocated to the cytosol (Young et al., 2002) possibly reflecting an mRNA regulation mechanism. PKCβII and PKCε then were seen in the membrane fraction at later time points suggesting a translocation back to the membrane coincident with the GAP-43 phosphorylation time course leading the authors to conclude that these two PKC isoforms were responsible for the phosphorylation of GAP-43.

Two studies reported on the phosphorylation state of GAP-43 and its effect on behavioral memory. Three lines of transgenic mice were developed (Aigner et al., 1995), each possessing an alteration of the PKC phosphorylation site on the GAP-43 amino acid sequence: (1) G-Phos mice overexpressed the native form (phosphorylatable and dephosphorylatable; no mutation) of chicken GAP-43; (2) G-Perm mice overexpressed chicken GAP-43 that was permanently, pseudophosphorylated with an aspartate substitution for serine at position 42; (3) the GNonP mice overexpressed a nonphosphorylatable form of chicken GAP-43 in which alanine was substituted at the serine-42 site. Assessment of behavioral memory function in each of these transgenic lines revealed the following: (1) G-Phos mice showed improved spatial memory function on a water maze task; (2) G-Perm mice showed a deficit in their ability to extinguish a classically-conditioned context-shock association indicative of a memory persistence; (3) G-NonP mice showed a significant impairment in their ability to recall spatial information on the water maze task (Holahan and Routtenberg, 2008). G-Phos mice were further subdivided based on their water maze acquisition rates and hippocampal GAP-43 levels into “spatial bright” and “spatial dull” groups. The “spatial dull” group showed impaired acquisition and retention on the spatial version of the water maze task but performed similar to controls on the visible platform task. The “spatial bright” group showed enhanced memory function compared to controls on a nonmatching to place water maze task (hidden platform moved every day). Interestingly, the “dull” group was found to have 50% more transgenic GAP-43 protein and a two-fold increase in transgenic GAP-43 mRNA in the hippocampus compared to the “bright” group (Holahan et al., 2007). These results indicated that elevated concentrations of the transgenic GAP-43 molecule impaired memory formation and subsequent studies (Rekart and Routtenberg, 2010) showed large aggregates in these mice reminiscent of protein aggregation as seen in neurodegenerative diseases (Jucker and Walker, 2013).

Two other studies examined the relationship between altered GAP-43 labeling and presynaptic remodeling after memory formation. Rats in the first study were given a spatial memory retention test 1 or 30-days after initial training. The 30-day group showed elevated GAP-43 staining in the anterior cingulate cortex compared to the 1-day retention group (Maviel et al., 2004). In the second study, high-resolution MRI analysis revealed a structural remodeling in the hippocampus of mice trained on the spatial version of a water maze task with correlated increased GAP-43 staining (Lerch et al., 2011).

### Critical or Supporting Factor in Plasticity?

An increase in presynaptic glutamate release has been shown to be associated with the observation of LTP (Lynch et al., 1990; Richter-Levin et al., 1995). Quantal analysis of failure rates before and after LTP induction in the hippocampus has revealed that enhanced synaptic efficacy is partially dependent on an increase in the probability of transmitter release (Bekkers and Stevens, 1990; Malinow and Tsien, 1990; Stevens and Wang, 1994). The influx of Ca^2+^ and binding to particular proteins involved in vesicular exo- and endocytosis mediates the transport and docking of glutamatergic vesicles facilitating vesicle recycling and release machinery. Because phosphorylated GAP-43 shows interactions with a number of synaptic vesicle proteins (Haruta et al., 1997), it may bridge the gap between those proteins involved in endocytosis and those involved in exocytosis (Rizo and Südhof, 2002; Sudhof, 2004; Jahn and Scheller, 2006; Sudhof and Rothman, 2009) thereby facilitating glutamate (or other neurotransmitters) vesicular recycling during times of plasticity including development, learning or regeneration. As a coordinator of axonal function, phosphorylation of GAP-43 would promote actin filaments not only for axonal rearrangement but also the coordinated release and recycling of transmitter vesicles (see Denny, 2006). In its contribution to vesicle recycling, phosphorylated GAP-43 would activate heterotrimeric G proteins translocating the protein to the presynaptic membrane and potentially leading to a change in membrane tension facilitating vesicular recycling (Denny, 2006). In addition to these actions, GAP-43 phosphorylation dissociates GAP-43 from CaM which would then interact with vesicular proteins. Phosphorylated GAP-43 by PKC would provide a sustained mechanism for vesicular recycling and release CaM to further enhance vesicular recycling leading to enhanced glutamate release, potentiation of cellular plasticity and enhanced memory function. In these cases, it appears that GAP-43 supports the activity of a number of proteins and kinases that are critical for presynaptic function.

## GAP-43 as A Mediator of Axonal Outgrowth

### Injury-Induced Regeneration and Associated Changes in GAP-43

Following axon damage, GAP-43 translation is up-regulated in close temporal sequence with nerve regeneration, potentially mimicking an early developmental phase (Benowitz and Routtenberg, 1997). As an example, a reduction in GAP-43 protein levels is noted in motor nerves and neuromuscular junctions during the second postnatal week. However, it is upregulated in these tissues during the injury-induced regeneration process and in mice overexpressing transgenic, native (phosphorylatable) GAP-43, nerve sprouting in these tissues is potentiated (Caroni, 1997). Likewise, overexpression of GAP-43 was reported to promote Purkinje axon plasticity and an enhanced regenerative potential even though, in the control condition, these neurons show poor regenerative potential (Rossi et al., 2001; Gianola and Rossi, 2004, 2005). Following laser axotomy of adult climbing fiber axonal branches, new branches with varicosities and a large numbers of vesicles were observed to sprout in close proximity to the intact surrounding Purkinje dendrites (Allegra Mascaro et al., 2013). Downregulation of GAP-43 mRNA activity via an interference approach resulted in a significant increase in the turnover of presynaptic boutons and impeded the appearance of reactive sprouts suggesting a critical role for GAP-43 in initiating and sustaining axonal regrowth after injury (Allegra Mascaro et al., 2013).

Three days after *in vivo* damage to the sciatic nerve in adult rats, GAP-43 staining begins to appear in the axotomized DRG cells with subsequent transport into the newly formed sprouts (Gispen et al., 1990; Woolf et al., 1990). GAP-43 staining intensity was observed to peak 21 days after damage and was absent 9 weeks following sciatic nerve crush injury and 36 weeks following nerve cut (Woolf et al., 1990). By using an RNA interference approach, GAP-43 downregulation resulted in a significant decrease in newly formed branches in climbing fibers following axotomy (Allegra Mascaro et al., 2013). In this report, GAP-43 mRNA downregulation also attenuated the appearance of newly formed sprouts indicating a causal link between GAP-43 and the initiation of axonal regrowth after injury (Allegra Mascaro et al., 2013). After spinal cord injury, administration of IL-6 was shown to enhance neurite outgrowth that was associated with an increased expression of GAP-43 mRNA indicating that IL-6 may be a factor that can initiate a GAP-43 associated growth outcome to promote axonal regrowth and functional recovery (Yang et al., 2012). In these instances, GAP-43 appears to play a critical role in axonal outgrowth.

Following deafferentation of the olfactory epithelium or after olfactory bulbectomy, levels of GAP-43 mRNA and protein increase in conjunction with the formation of immature olfactory sensory neurons (Verhaagen et al., 1990). Three weeks after binocular retinal lesions (central 10 degrees) in adult cats, GAP-43 staining was elevated in the dorsal lateral geniculate nucleus region that represents this retinal area (Baekelandt et al., 1994). Elevated GAP-43 staining was also found in catecholaminergic and serotonergic axonal sprouts that regenerate around the damaged area associated with axotomy of the medial forebrain bundle (Alonso et al., 1995). Cochlear neuron removal in adult rats results in elevated GAP-43 protein staining in the fibers of the ipsilateral ventral cochlear nucleus and somas of the lateral superior olive (Illing and Horvath, 1995). After unilateral cochlear ablation, GAP-43 expression was tightly linked with the accumulation of matrix metalloprotease-2 (MMP-2), a major actor in extracellular matrix remodeling suggesting that MMP-2 may provide a signal for GAP-43-directed axonal outgrowth and synaptogenesis following damage (Fredrich and Illing, 2010). Five to 7 days after experimental tooth movement, axonal terminals of the periodontal Ruffini endings showed elevated GAP-43 staining with a complete disappearance 14 days later suggesting that GAP-43 may function as a key molecule in the remodeling of mechanoreceptive endings during tooth movement (Kobayashi et al., 1998).

In an animal model of middle cerebral artery occlusion (MCAo), behavioral recovery as assessed with a pellet-reaching task occurred during a 14-day period after MCAo and in the affected cervical gray matter, GAP-43 staining on corticospinal tract axons was significantly increased during the same period while synaptophysin increased in axonal terminals at 28 days (Liu et al., 2013). In another stroke model, optogenetic stimulation in the primary motor cortex on the same side of the damage was shown to facilitate motor recovery and increase the expression of multiple neurotrophins such as brain-derived neurotrophic factor (BDNF), NGF as well as increase in the expression of GAP-43 (Cheng et al., 2014).

GAP-43 immunoreactivity in layer IV of the barrel cortex receptor field are moderate in the inter-barrel septa and low within the barrels themselves in the adult rat (Dunn-Meynell et al., 1992). The distribution of GAP-43 was then examined 1–8 weeks after unilateral vibrissectomy of all but the C3 vibrissa to assess the outcome and time course on GAP-43 distribution in the barrel cortex. Following vibrissectomy, the GAP-43 immunonegative C3 area showed a reduction in volume from 8.4% at 1 week after injury to a 12% decrease 8 weeks after damage relative to the control ipsilateral cortex suggesting a GAP-43-mediated axonal sprouting within the barrel cortex whereby GAP-43 positive terminals encroached on areas that lacked GAP-43 (Dunn-Meynell et al., 1992). In a second study utilizing a similar injury model (unilateral vibrissectomy sparing the C3 vibrissa), GAP-43 levels were elevated 25% compared to the unlesioned side for 6 days following surgery then decreased by 88% at 7 days and returned to baseline by 14 days (Levin and Dunn-Meynell, 1993).

Lesions of the hippocampal perforant path are associated with an elevated distribution of GAP-43 protein during the phase of synaptogenesis (Neve et al., 1991). Following unilateral entorhinal cortex (EC) lesions in adult rats, a 2-fold (100%) increase of recently synthesized GAP-43 was noted in the contralateral hippocampus where sprouting occurred. This surge in GAP-43 mobilization happened from 6 to 15 days after the lesion and coincided with the growth of presynaptic terminals (Lin et al., 1992).

In post-mortem AD brains, GAP-43 labeling indicated presynaptic neurite outgrowth in the hippocampal molecular layer, *stratum polymorphous*, the CA1 and prosubiculum with a high number of GAP-43-immunoreactive coiled fibers and dystrophic neurites closely apposed to plaques (Masliah et al., 1991). A reliable increase in GAP-43 staining in the *stratum lacunosum moleculare* subfield of the hippocampus was also observed in AD patients compared to age-matched controls and was positively correlated with the severity of AD (Rekart et al., 2004). This hippocampal subfield contains inputs from the EC so the authors surmised that increased GAP-43 expression reflected aberrant neuronal sprouting and this, in conjunction with neurodegeneration, could culminate in memory dysfunction associated with AD (Rekart et al., 2004).

In addition to physical structural damage resulting in elevated GAP-43 levels and axonal sprouting, excitotoxicity has been shown to increase GAP-43-dependent sprouting (Gravel et al., 2011). In one such study (Cantallops and Routtenberg, 1996), 12 h after subcutaneous administration of the excitatory agonist kainate, GAP-43 mRNA expression increased in granule cells and 2 days after kainate injection (up to 40 days), GAP-43 protein levels and mossy fiber sprouting into the supragranular layer of the hippocampus were observed. These outcomes are similar to those seen after structural or neuronal damage which result in axonal outgrowth with one difference that kainate treatment did not result in substantial physical cell loss (Cantallops and Routtenberg, 1996). The sum total of these studies suggests that elevations in either GAP-43 mRNA or protein levels or, enhanced phosphorylation status of GAP-43 lead to elevated axonal structural remodeling thereby revealing a critical role for this growth-associated factor.

### GAP-43 Stabilization as a Critical Factor in Axonal Outgrowth

Because HuD protein serves to stabilize GAP-43 mRNA, it may be one target to enhance GAP-43 activity and hence, promote axonal outgrowth. Overexpression of HuD protein in PC12 cells stabilized GAP-43 mRNA by attenuating the onset of mRNA degradation (Beckel-Mitchener et al., 2002) and morphological analysis revealed multiple GAP-43-positive neurites that contained tubulin and F-actin that occurred in the absence of NGF with overexpression of full length but not truncated HuD (Anderson et al., 2000). With truncated HuD, GAP-43 mRNA was not stabilized and these cells showed no spontaneous outgrowth and a lack of growth in the presence of NGF (Anderson et al., 2000). While in normal cortical neurons, *in vitro* neurite outgrowth occurs over a 3-day period with associated elevations in GAP-43 and HuD expression; overexpression of HuD can accelerate the formation of neurite outgrowth, increase the number of GAP-43 positive cells that undergo differentiation and result in a twofold increase in GAP-43 mRNA (Anderson et al., 2001). These results point to a central role for HuD regulation of GAP-43 expression during the initial stages of neurite outgrowth (Anderson et al., 2001) and may therefore be a target for jump-starting a therapeutic process in regeneration after injury. In this regard, the levels and expression of HuD and GAP-43 mRNA in DRG neurons were examined following sciatic nerve injury (Anderson et al., 2003). Seven days following injury, HuD and GAP-43 mRNA in the ipsilateral DRG increased by two- and six-fold relative to the contralateral DRG (Anderson et al., 2003). That GAP-43 stability may be the pivotal factor in facilitated neurite outgrowth comes from evidence that overexpression of full length KH-type splicing regulatory protein (KSRP), which promotes the deterioration of GAP-43 mRNA, hindered axonal outgrowth in hippocampal cultures while depletion of KSRP led to a rise in GAP-43 mRNA levels and a significant increase in axonal length (Bird et al., 2013). Overexpression of GAP-43 was shown to reverse the axonal outgrowth inhibition seen with KSRP overexpression, but only when the cells were transfected with GAP-43 that was targeted for transport to the axons (Bird et al., 2013). These results point to KSRP as a central regulator of GAP-43 mRNA stability and axonal malleability that works in contrast to HuD in the regulation of GAP-43 neuronal mRNAs (Bird et al., 2013). Therefore, enhancing the stability of GAP-43 mRNA via mRNA binding proteins, such as HuD, may facilitate intracellular protein-protein interactions culminating in the appropriate intracellular environment for axonal elongation (van Kesteren et al., 2011).

### Targeting the Inhibitors of GAP-43

Another potential, indirect mechanism for elevating GAP-43 expression and facilitating neurite outgrowth is via inhibition of Nogo-A-dependent processes. Nogo-A is a myelin-derived inhibitor that plays a central role in the prevention of axonal outgrowth (Chen et al., 2000; Pot et al., 2002). Axons within the central nervous system show poor potential to recover after an insult due to the actions of myelin-derived inhibitors of axonal outgrowth such as Nogo, myelin-associated glycoprotein (MAG) and oligodendrocyte-myelin glycoprotein (OMgp), all of which show affinity and bind to the Nogo-66 receptor (for reviews, see Huber and Schwab, 2000; Reilly, 2000; Fournier et al., 2002; Pernet and Schwab, 2012; Schwab and Strittmatter, 2014). In order for GAP-43 to be effective in coordinating axonal regeneration, it might be necessary to inhibit the action of Nogo or other inhibitors of axonal growth.

In two studies examining axonal repair after traumatic brain injury (TBI), inhibiting Nogo-A expression with an anti-Nogo-A monoclonal antibody (Marklund et al., 2007) or with exercise (Chytrova et al., 2008) resulted in an upregulation of GAP-43 as well as improved functional outcomes during the regenerative period. Following damage in the rat corticospinal tract, application of the anti-Nogo-A monoclonal antibody was associated with upregulation of GAP-43 and triggered regenerative sprouting across the midline with appropriate sensory and motor fiber innervation that was stable and persistent (Bareyre et al., 2002). Axonal regrowth emanating from retinal ganglion cells after optic nerve damaged was investigated in both Nogo-A knockout mice (Su et al., 2008) and Nogo-66 receptor knockout mice (Su et al., 2009). In both cases, GAP-43 expression was significantly higher during the regeneration period and axonal growth was significantly greater than the wild type condition. In primary retinal ganglion cells, Nogo-A RNA inhibition or a peptide antagonist of the Nogo-66 functional domain promoted axonal outgrowth as assessed using GAP-43 immunofluorescence (Huo et al., 2013) suggesting a close relationship between GAP-43 and neurite extension induced by inhibition of Nogo-A function. In rats with spinal cord hemisection, adenovirus-mediated transfection of Nogo-A short hairpin RNAs were found to reduce the expression of the Nogo-A gene and upregulate GAP-43 expression with an associated functional recovery of the injured nerves (Liu et al., 2016). Therefore, impeding Nogo gene expression may be an important step leading to the upregulation of GAP-43 in the promotion of spinal cord axonal outgrowth. In this case, Nogo would be the critical factor in axonal outgrowth and GAP-43 would support this function.

Systemic administration of small interfering RNA-Nogo-A in a mouse model of experimental autoimmune encephalomyelitis was shown to enhance GAP-43 positive axons in the lesioned area and promote axonal repair (Yang et al., 2010). Four weeks after TBI, scores on a hippocampal-dependent novel object recognition test were increased in Nogo-66 knockout mice compared to wild-type along with elevated GAP-43 protein staining in the injured and contralateral sides of the hippocampus (Tong et al., 2013). In a similar TBI model, progesterone administration was shown to significantly decrease the expression of Nogo-A and upregulate GAP-43 protein suggesting that progesterone may promote neuroprotection by inhibiting Nogo-A and increasing GAP-43 (Liu et al., 2014). Constraint-induced movement therapy after focal cerebral ischemia in rats was reported to significantly increase the length and number of midline crossings of corticospinal axons, decreased the expression of Nogo-A/Nogo receptor and increase the expression of GAP-43 in the denervated spinal cord all associated with improved motor function (Zhao et al., 2013). Therefore, the synergistic effect of reducing Nogo-A-inhibitory processes and upregulating intracellular GAP-43 growth permissive processes may provide the optimal environment for axonal regeneration. In this scenario, GAP-43 would be the supporting factor mediating a critical step between extracellular signals and intercellular membrane dynamics.

## Concluding Remarks

The historical and current work investigating the role of GAP-43 in nervous system function has revealed that its mRNA expression or protein levels increase in conjunction with axonal structural plasticity as occur during development and maturation, with input-dependent memory related processes and following axonal injury during the regeneration phase. Because all of these events can culminate in reorganization of presynaptic elements (i.e., axons), the transcriptional and post-transcriptional regulation of the mRNA and the post-translational modification (phosphorylation) and protein localization were intensely scrutinized as a means to facilitate central nervous system plasticity. Many growth permissive events in the central nervous system are associated with the augmentation of GAP-43 levels suggesting it may have been a critical factor for plasticity-associated axonal function. While upregulation of GAP-43 protein phosphorylation status, enhanced stability of GAP-43 mRNA and inhibition of Nogo-A/Nogo66 inhibitory processes have all been shown to promote axonal outgrowth, GAP-43 appears to play a more permissive role in axonal plasticity being an important node in coordinating various other critical factors. If nothing else, the exploration of GAP-43 function and upstream and downstream regulators has stimulated the search for targeted therapeutics directed to facilitate CNS regeneration after axonal injury (see Ribas and Costa, 2017) and possibly, developmental disorders, memory dysfunction and neurodegenerative diseases.

## Author Contributions

MRH wrote and edited the review manuscript and is responsible for all content.

## Conflict of Interest Statement

The author declares that the research was conducted in the absence of any commercial or financial relationships that could be construed as a potential conflict of interest.
